# Author Correction: H3.3 deposition counteracts the replication-dependent enrichment of H3.1 at chromocenters in embryonic stem cells

**DOI:** 10.1038/s41467-026-70009-x

**Published:** 2026-02-26

**Authors:** Stefano Arfè, Tina Karagyozova, Audrey Forest, Dominic Bingham, Hatem Hmidan, David Mazaud, Mickaël Garnier, Patricia Le Baccon, Eran Meshorer, Jean-Pierre Quivy, Geneviève Almouzni

**Affiliations:** 1 Nuclear Dynamics, Institut Curie, PSL University, Sorbonne Université, CNRS, Paris, France; 2 Department of Genetics, The Alexander Silberman Institute for Life Science, and the Edmond and Lily Safra Center for Brain Sciences (ELSC), The Hebrew University of Jerusalem, Jerusalem, Israel; 3 Present Address: Center for Neural Science and Medicine, Department of Biomedical Sciences, Cedars-Sinai Medical Center, Los Angeles, CA USA; 4 Present Address: Physiology and pharmacology department, college of medicine, Al-Quds University, Jerusalem, Palestine; 5 Present Address: School of Biological Sciences, Institute of Cell Biology, University of Edinburgh, Edinburgh, UK

**Keywords:** Epigenetics, Embryonic stem cells

Correction to: *Nature Communications* 10.1038/s41467-025-60430-z, published online 3 June 2025

In the version of this article initially published, all authors were listed with initials instead of full first names and this has now been updated in the HTML and PDF versions of the article.

