# Supplementary material for: H3.3 deposition counteracts the replication-dependent enrichment of H3.1 at chromocenters in embryonic stem cells
Source: Nat Commun. 2025 Jun 3;16:5138. doi: 10.1038/s41467-025-60430-z (PMC12134209; doi:10.1038/s41467-025-60430-z)

H3.3 deposition counteracts the replication-dependent enrichment of H3.1 at chromocenters in embryonic stem cells

S. Arfè [1,3], T. Karagyozova [1,5], A. Forest [1], D. Bingham [1], H. Hmidan [1,4], D. Mazaud [1], M. Garnier [1], P. Le Baccon [1],E. Meshorer [2], J.-P. Quivy [1*] and G. Almouzni [1*]

[1] Institut Curie, PSL University, Sorbonne Université, CNRS, Nuclear Dynamics, 75005 Paris, France

[2] Department of Genetics, The Alexander Silberman Institute for Life Science, and the Edmond and Lily Safra Center for Brain Sciences (ELSC), The Hebrew University of Jerusalem, Jerusalem, Israel 9190400.

[3] Present address: Center for Neural Science and Medicine, Department of Biomedical Sciences, Cedars-Sinai Medical Center, Los Angeles, CA 90048, USA

[4] Present address: Physiology and pharmacology department, college of medicine, Al-Quds University, Jerusalem, Palestine.

[5] Present address: School of Biological Sciences, Institute of Cell Biology, University of Edinburgh, Edinburgh, EH9 3FF, UK

* Corresponding authors:

Genevieve.almouzni@curie.fr

jean-pierre.quivy@curie.fr

Supplementary Information

Supplementary informations are provided as a single .pdf file with the following contents:

- Supplementary figures S1 to S6 legends
- Supplementary figures S1 to S6
- scans of blots figure S1

Supplementary figure legend

Supplementary Figure 1 (related to Figure 1). H3-SNAP-Tagged histones are all expressed and incorporated in a similar mode *in vivo*. (**a**) Schematic and strategy to introduce H3.1 or H3.3 histone variants in mouse ESCs. H3.1 (or H3.3) CDS is fused upstream to a SNAP-Tag-3x-HA sequence and cloned into an expression vector that is stably integrated downstream of the Type I Collagen (Col1A1) locus containing a Frt site under the control of a TET-ON (Dox-inducible) regulatory region. (**b**) Analysis of expression of SNAP-3xHA H3 variants by Western blot of total proteins in ESCs and differentiated NPCs. The presence of SNAP-tagged histones is verified with HA antibody. Differentiation from ESCs to NPCs is verified via Oct3/4 expression. (**c**) Representative epifluorescence images of H3.1-SNAP (top) and H3.3-SNAP (bottom) detected by TMR labeling of SNAP (red) in ESCs after various times of exposure to Dox. DNA (DAPI) is shown in cyan. Scale bar 5 μm. (**d**) Comparison by Western blot of endogenous and exogenous expression of H3 histones in ESCs, NPCs, and NIH-3T3 cells. Total protein extracts from 1*10^6 cells (= x) are loaded per lane. The presence of H3 variants is verified by H3.1/2 and H3.3 specific antibodies on two separate membranes at different time exposures. Tagged and endogenous histones are reported at their corresponding heights. H4 histone is used as a loading control. * = non-specific bands. Scale bars. (**e**) Top: representative epifluorescence images of 3 nuclei displaying ‘enriched’, ‘even’, and ‘excluded’ patterns for H3.1-SNAP (left) and H3.3-SNAP (right) visualized by TMR labeling of SNAP (red) ESCs along with ~~and~~ DNA (DAPI, cyan). Scale bar is 10 μm. The dotted line over a chromocenter indicates the scan-line used below. Bottom: scan line profiles of H3 from the above chromocenters displaying ‘enriched’, ‘even’, and ‘excluded’ patterns for H3.1-SNAP and H3.3-SNAP (red) along with corresponding DAPI (blue). The profile of each chromocenter

was normalized to 1 and 0 for the max and min value. The curve show the mean of the 3 normalized profiles and bars the s.e.m.

Supplementary Figure 2 (related to Figure 1). H3.1/2 is strongly associated with mouse chromocenters during differentiation. (**a**) Consecutive z-series of confocal images (Nikon AXR; z-step 0.172 μm) showing endogenous H3.1/2 (Red) and H3.3 (Green) co-stained in ESCs along with DNA counterstaining (DAPI, cyan). Arrow heads point to chromocenters displaying H3.1/2 enrichment and H3.3 depletion. Scale bar, 10 μm. (**b**) Representative epifluorescence images of H3.1- (or H3.3) SNAP (red) in mouse ESCs, NPCs, or NIH-3T3 along with HP1α (green) and DNA (DAPI, cyan). Clusters of pericentric heterochromatin (PHC) were identified as DAPI-dense domains. (**c**) Boxplots of H3.1/2 and H3.3 enrichment at chromocenters in ESCs (72 nuclei) and NIH-3T3 cells (86 nuclei). The centre of the boxplot is the median, the bounds of the box are the 1st and 3rd quartiles and the whiskers extend to max and min. (**d**) Scheme for SNAP-Seq in mESCs and NPCs expressing H3.1- or H3.3- SNAP. MNase digestion optimized to obtain mononucleosomes next subjected to capture and pull-down with SNAP beads, followed by sequencing.

Supplementary Figure 3 (related to Figure 3). H3.3-oncohistones are not enriched at chromocenters. Quantification of cells expressing H3 oncohistones and exhibiting H3 patterns at PHC during Early, Mid, and Late S stages. Stacked histograms show the mean (in %) and bars s.d from 2 experiments. n= 143 for K27M; 148 for K27L; 154 for G34R; 156 for G34V.

Supplementary Figure 4 (Related to figure 4). Targeting of HIRA to chromocenters leads to increased detection of H3.3 at chromocenter. Confocal images (Zeiss LSM 780) showing co-staining of endogenous H3.1/2 (green) and H3.3 (red) along with Clover (magenta) and

DAPI (cyan) in control non-transfected (NT), Ctr, TALE wt and TALE mut as indicated. Merge image of H3.1/2 and H3.3 is shown on the right. Scale bar is 5 µm. Dashed line boxes highlight a chromocenter in each image. The column on the right displays A zoom of the chromocenter from the merge chanel is shown with scale bar 1 µm.

Supplementary Figure 5 (related to figure 4). HIRA targeting at PHC does not alter Major Satellite transcription at chromocenters and CENP-A localization. (**a**) Representative epifluorescent images of situ Major Satellite RNA (FISH) (MajSat RNA, red) along with Clover reporter (green) and DNA (DAPI, cyan) in cells expressing Ctr, HIRA wt and HIRA mut. Scale bar 10 um. (**b**) Quantitative analysis of the TALE constructs expressing ESCs exhibiting MajSat RNA foci. Bars display the mean and s.d from 3 experiments for HIRA wt and 2 experiments for Ctr and HIRA mut. n=66, for Ctr; 139 for TALE wt; 69 for TALE mut (**c**) Quantitative analysis of the number of MajSat RNA foci detected per nucleus TALE constructs expressing ESCs. Bars display the mean and s.d from 3 experiments for HIRA wt and 2 experiments for Ctr and HIRA mut. n=46 for Ctr; 89 for HIRA wt; 49 for TALE-HIRA mut. **(d)** Representative immunofluorescent images of ESCs transfected with Ctr, HIRA wt and HIRA mut TALE constructs. Clover (green) is detected specifically at chromocenters, along with CENP-A (red) antibody, and DNA (DAPI, cyan) staining. Scale bar, 10 µm.

Supplementary Figure 6 (related to Fig. 5). HIRA targeting at PHC does not alter Major Satellite transcription at chromocenters. (**a**) Representative epifluorescent images of altered or unaltered ES cell nuclei upon HIRA wt TALE construct transfection. DAPI (cyan) and TALE (green) visualized by Clover fluorescence are shown. Scale bar, 10 µm. (**b**) Quantitative analysis of the percentage of abnormal nuclei in transfected cells from 6 experiments. Bar plots show average and standard deviations from 6 experiments. n=470 for Ctr, 554 for HIRA wt and

261 for Hira mut. Unpaired T test was used. *** = p value <0.001. P values Ctr vs HIRA wt = 0.00010; HIRA wt vs HIRA mut = 0.00045. Source data are provided as a Source Data file.

Figure S1

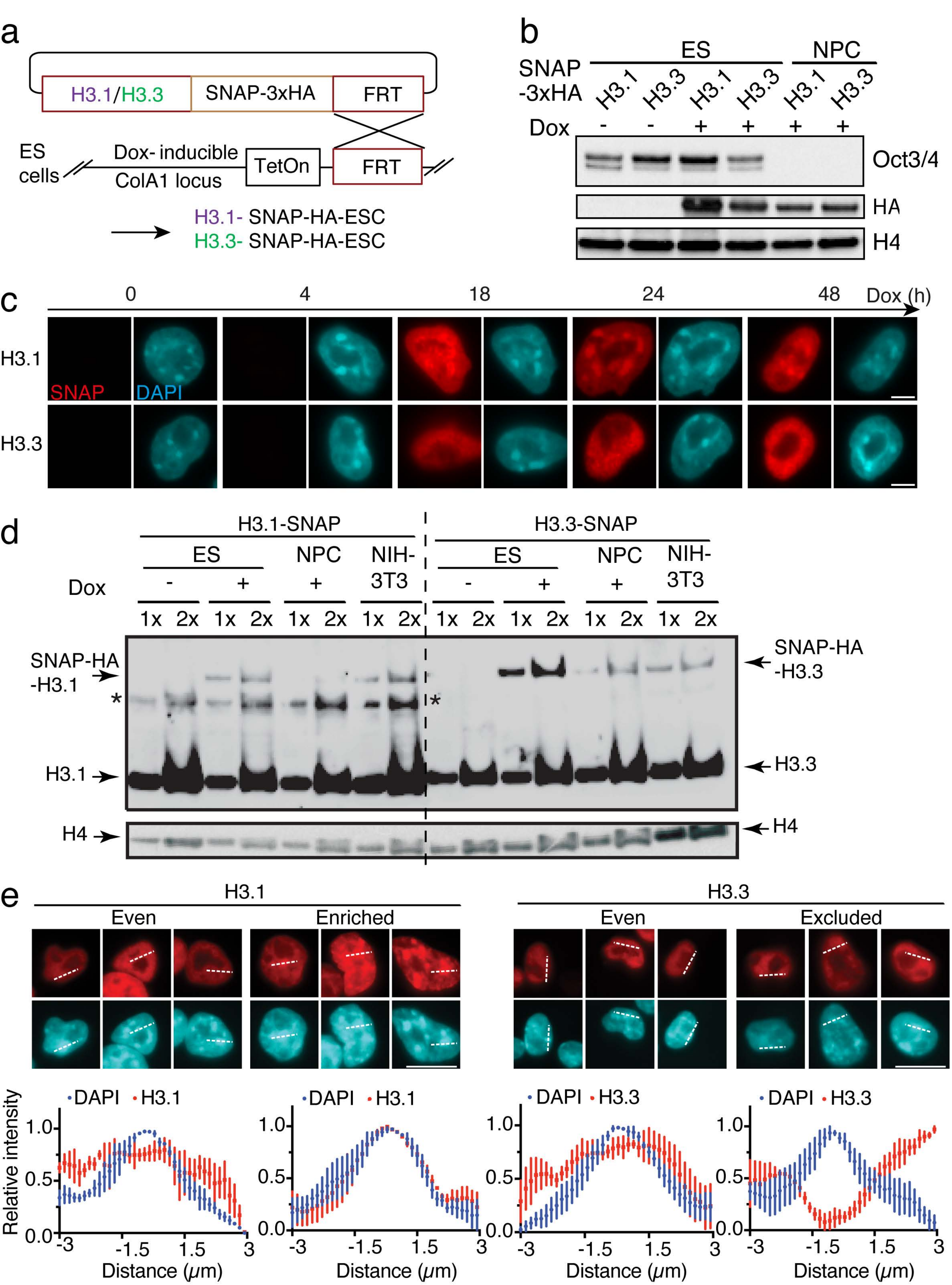
a
H3.1/H3.3
SNAP-3xHA
FRT
ES cells
Dox- inducible
ColA1 locus
TetOn
FRT
H3.1- SNAP-HA-ESC
H3.3- SNAP-HA-ESC
b
SNAP -3xHA
ES
NPC
H3.1 H3.3 H3.1 H3.3 H3.1 H3.3
Dox - - + + + +
Oct3/4
HA
H4
c
0 4 18 24 48 Dox (h)
H3.1
H3.3
SNAP
DAPI
d
H3.1-SNAP
H3.3-SNAP
ES
NPC
NIH-3T3
Dox - + +
1x 2x
SNAP-HA -H3.1
SNAP-HA -H3.3
*
H3.1
H3.3
H4
e
H3.1
H3.3
Even
Enriched
Excluded
DAPI H3.1
DAPI H3.3
Relative intensity
1.0
0.5
0.0
-3 -1.5 1.5 3
Distance (μm)

Figure S2

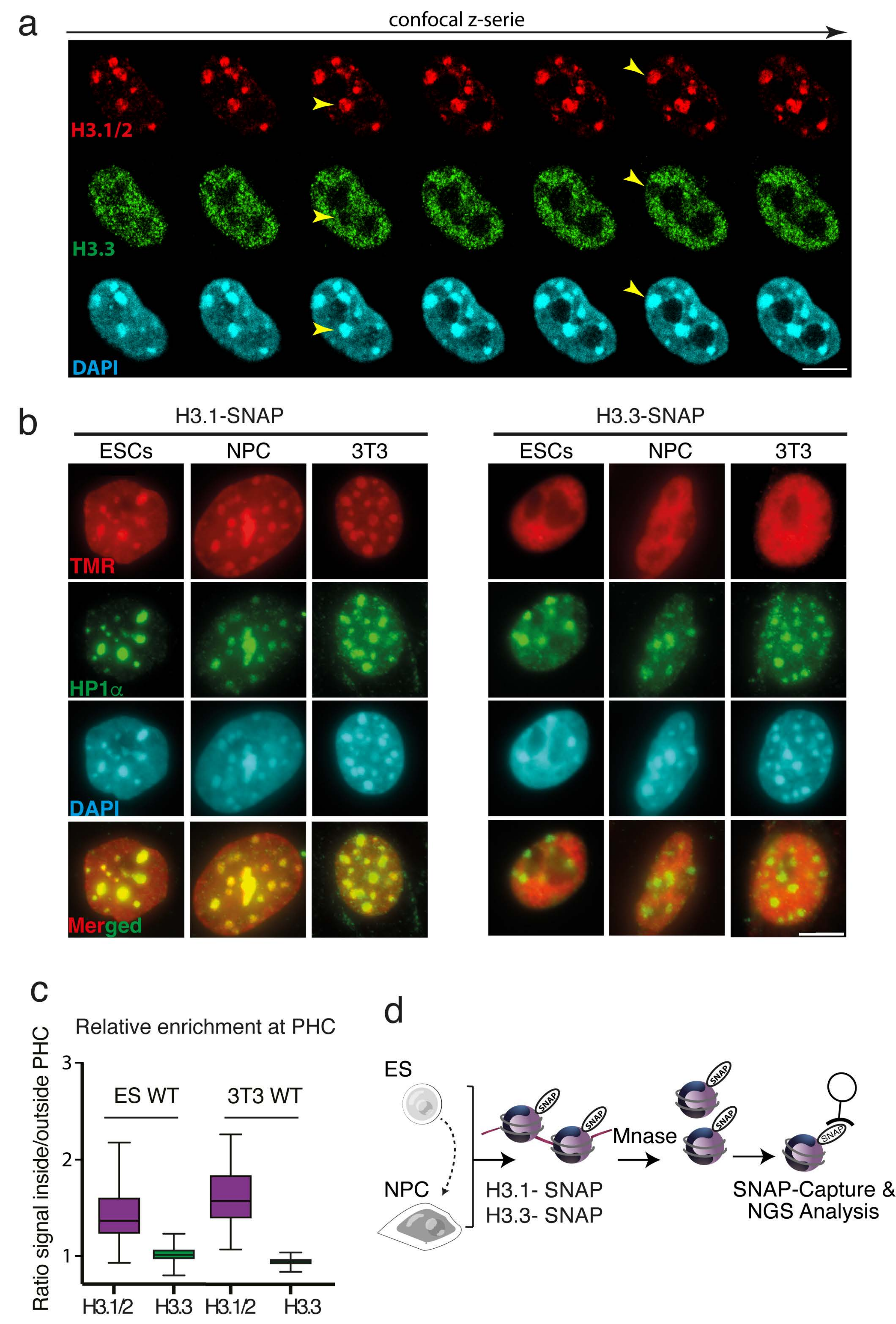
a
confocal z-serie
H3.1/2
H3.3
DAPI
b
H3.1-SNAP
H3.3-SNAP
ESCs
NPC
3T3
TMR
HP1α
DAPI
Merged
c
Relative enrichment at PHC
Ratio signal inside/outside PHC
ES WT
3T3 WT
3
2
1
H3.1/2
H3.3
d
ES
NPC
SNAP
Mnase
H3.1- SNAP
H3.3- SNAP
SNAP-Capture &
NGS Analysis

Figure S3

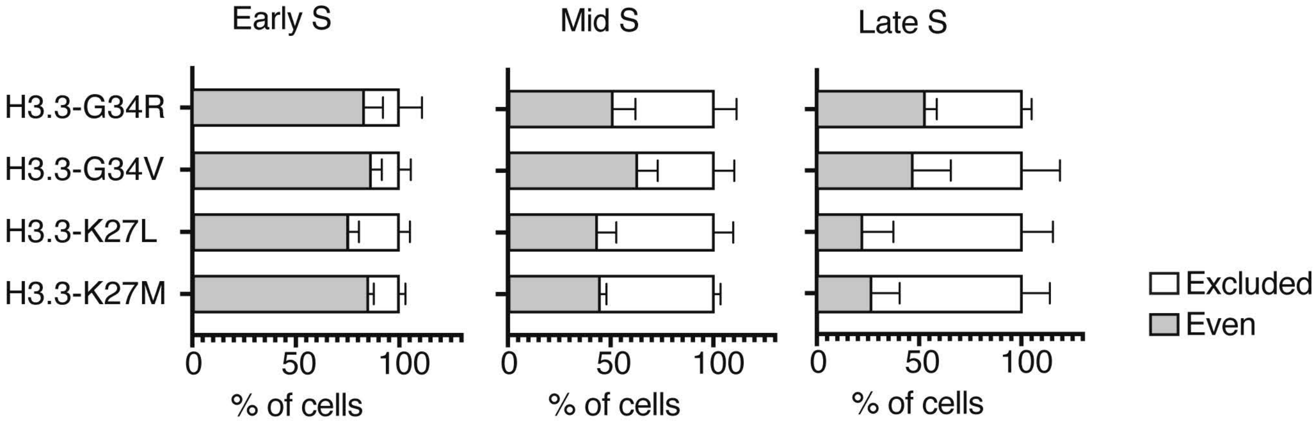

Early S
Mid S
Late S
H3.3-G34R
H3.3-G34V
H3.3-K27L
H3.3-K27M
0
50
100
% of cells
Excluded
Even

Figure S4

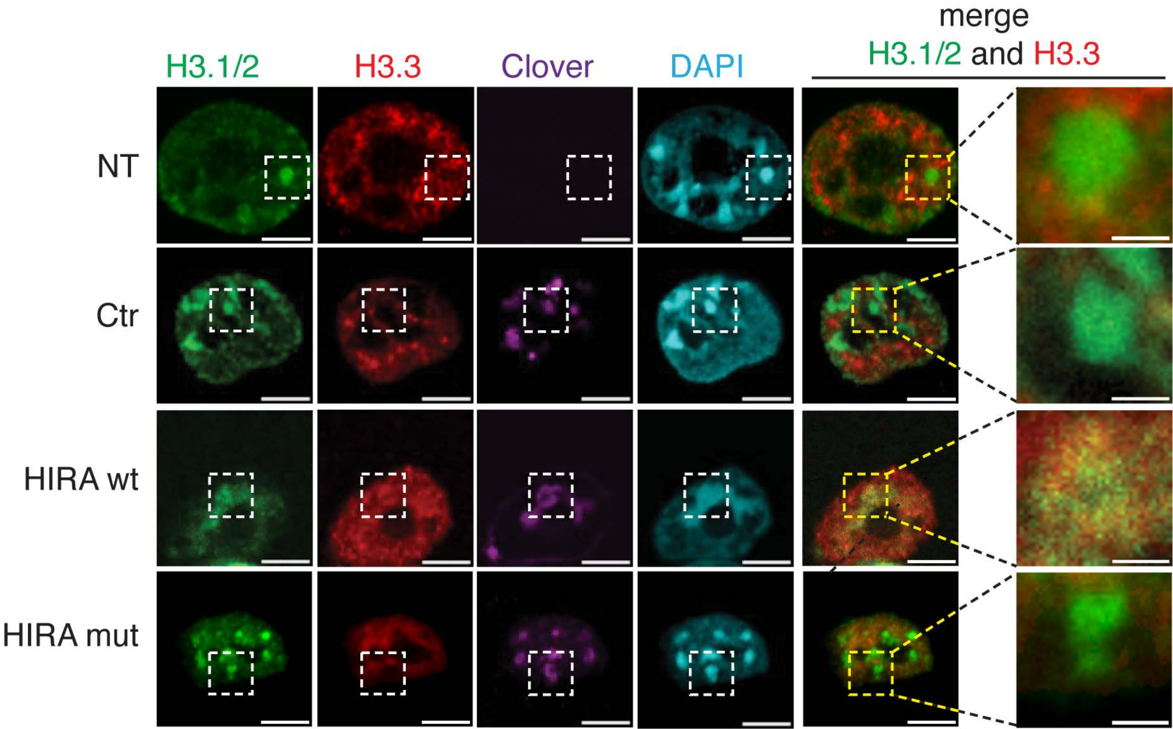

merge
H3.1/2 and H3.3
H3.1/2
H3.3
Clover
DAPI
NT
Ctr
HIRA wt
HIRA mut

Figure S5

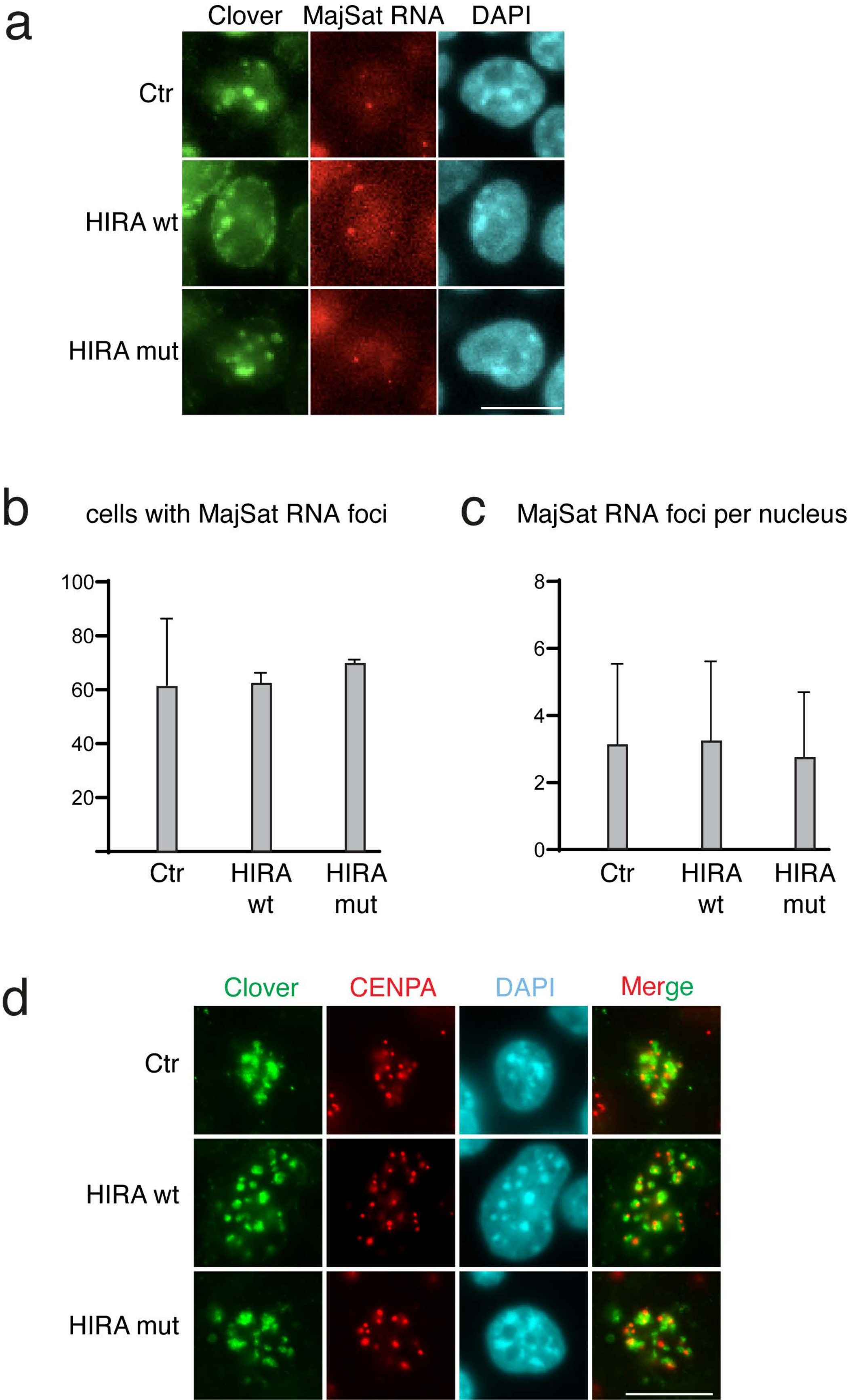

a
Clover
MajSat RNA
DAPI
Ctr
HIRA wt
HIRA mut
b
cells with MajSat RNA foci
100
80
60
40
20
Ctr
HIRA wt
HIRA mut
c
MajSat RNA foci per nucleus
8
6
4
2
0
Ctr
HIRA wt
HIRA mut
d
Clover
CENPA
DAPI
Merge
Ctr
HIRA wt
HIRA mut

Figure S6

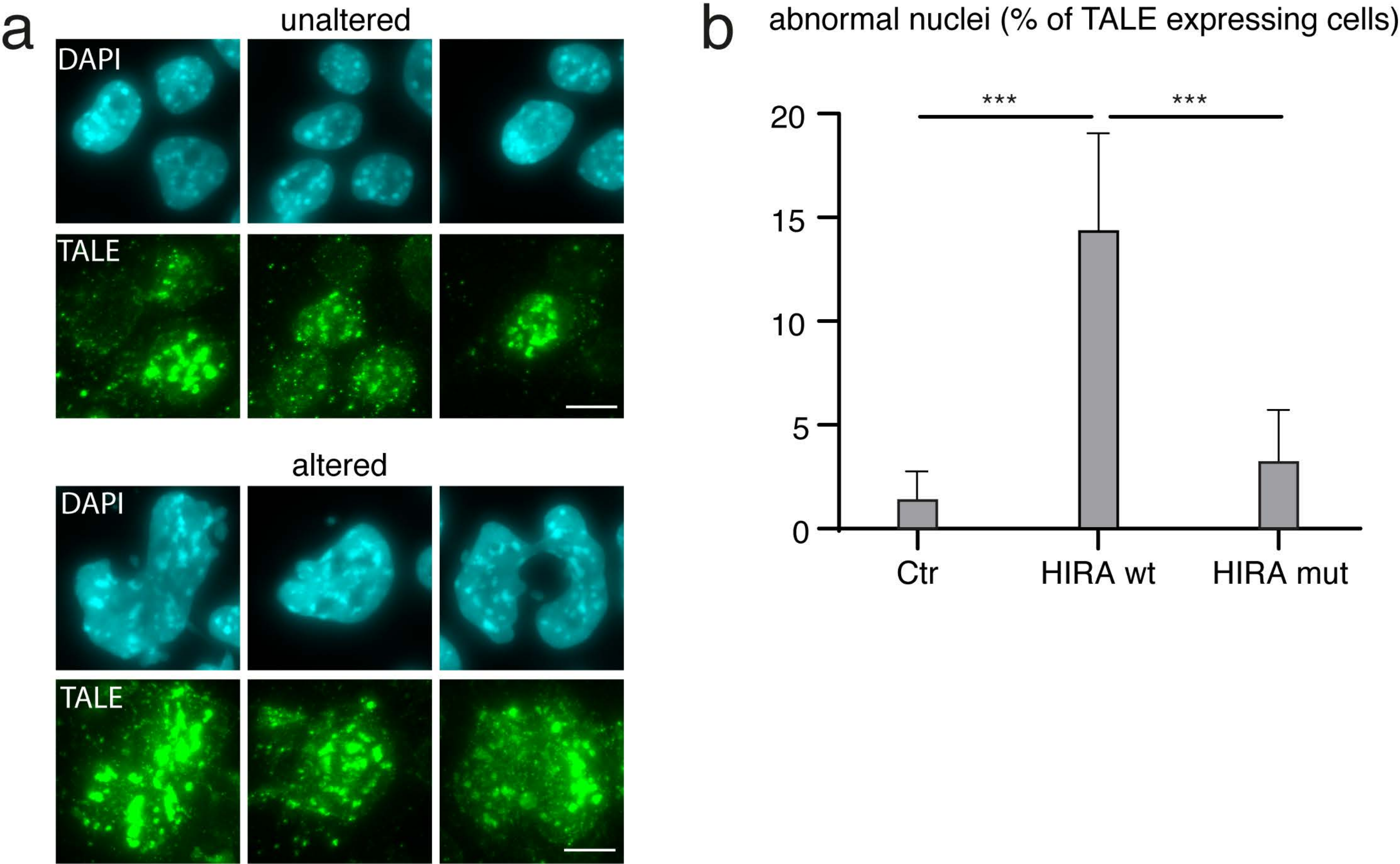
a
unaltered
DAPI
TALE
altered
DAPI
TALE
b
abnormal nuclei (% of TALE expressing cells)
***
***
20
15
10
5
0
Ctr
HIRA wt
HIRA mut

Source data: uncropped scans figure S1

b

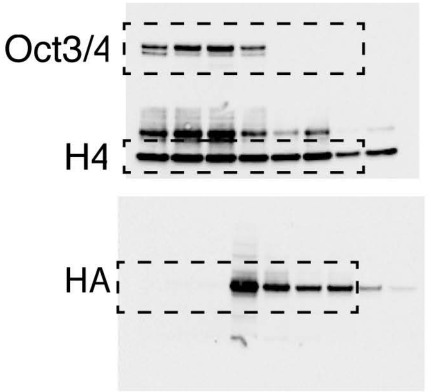

d

Membranes after every detections

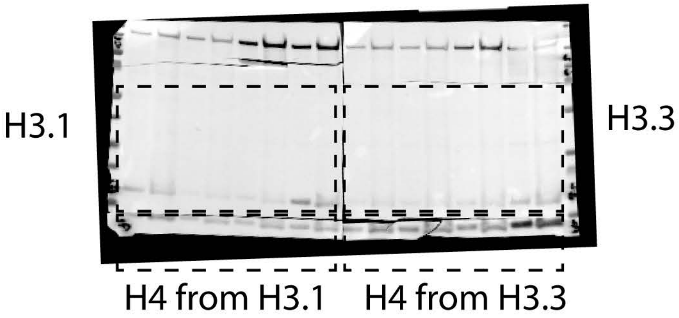

Immunodetection

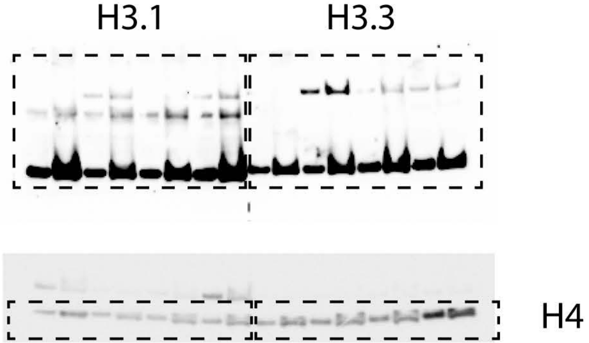

Supplement: Supplementary file 1 — Supplementary Information [file 41467_2025_60430_MOESM1_ESM.pdf]
