# Supplementary material for: H3.3 deposition counteracts the replication-dependent enrichment of H3.1 at chromocenters in embryonic stem cells
Source: Nat Commun. 2025 Jun 3;16:5138. doi: 10.1038/s41467-025-60430-z (PMC12134209; doi:10.1038/s41467-025-60430-z)
Supplement: Supplementary file 2 — Reporting Summary [file 41467_2025_60430_MOESM2_ESM.pdf]

nature portfolio

Corresponding author(s): Almouzni Genevève

Last updated by author(s): April 25 2025

Reporting Summary

Nature Portfolio wishes to improve the reproducibility of the work that we publish. This form provides structure for consistency and transparency in reporting. For further information on Nature Portfolio policies, see our Editorial Policies and the Editorial Policy Checklist.

Statistics

For all statistical analyses, confirm that the following items are present in the figure legend, table legend, main text, or Methods section.

n/a	Confirmed	
☐	☒	The exact sample size (*n*) for each experimental group/condition, given as a discrete number and unit of measurement
☐	☒	A statement on whether measurements were taken from distinct samples or whether the same sample was measured repeatedly
☐	☒	The statistical test(s) used AND whether they are one- or two-sided<br>*Only common tests should be described solely by name; describe more complex techniques in the Methods section.*
☒	☐	A description of all covariates tested
☒	☐	A description of any assumptions or corrections, such as tests of normality and adjustment for multiple comparisons
☐	☒	A full description of the statistical parameters including central tendency (e.g. means) or other basic estimates (e.g. regression coefficient) AND variation (e.g. standard deviation) or associated estimates of uncertainty (e.g. confidence intervals)
☐	☒	For null hypothesis testing, the test statistic (e.g. *F*, *t*, *r*) with confidence intervals, effect sizes, degrees of freedom and *P* value noted<br>*Give P values as exact values whenever suitable.*
☒	☐	For Bayesian analysis, information on the choice of priors and Markov chain Monte Carlo settings
☒	☐	For hierarchical and complex designs, identification of the appropriate level for tests and full reporting of outcomes
☒	☐	Estimates of effect sizes (e.g. Cohen's *d*, Pearson's *r*), indicating how they were calculated

Our web collection on statistics for biologists contains articles on many of the points above.

Software and code

Policy information about availability of computer code

Data collection	No software was used in this study
Data analysis	We used Fiji software for microscopy image visualization and time lapse analysis . For STORM microscopy, the drift correction was done using the Neo in-built RCC cross-correlation method with 1500 frames stack size and 25 nm lateral pixel size. A final DME algorithm (Cnossen, 2021 DOI: 10.1364/OE.426620) with an in-house adapted version in Matlab2024b using 50 frames as parameter was applied. STORM image rendering was done using 10 nm pixel size.<br>For SNAP-Capture Seq analysis (ChIP-Seq) we mapped reads to the soft-masked mouse reference genome (GRCm38, release 98) from Ensembl with bowtie2 v2.3.4.2 (Langmead, 2012 DOI: 10.1038/nmeth.1923-very-sensitive option). We sorted, flagged duplicates and indexed bam files with SAMtools v1.9 (Danecek, 2021 DOI: 10.1093/gigascience/giab008). This is available in text

For manuscripts utilizing custom algorithms or software that are central to the research but not yet described in published literature, software must be made available to editors and reviewers. We strongly encourage code deposition in a community repository (e.g. GitHub). See the Nature Portfolio guidelines for submitting code & software for further information.

Data

Policy information about availability of data

All manuscripts must include a data availability statement. This statement should provide the following information, where applicable:

- Accession codes, unique identifiers, or web links for publicly available datasets
- A description of any restrictions on data availability
- For clinical datasets or third party data, please ensure that the statement adheres to our policy

Sequencing data from SNAP capture assays have been deposited in ArrayExpress under accession number E-MTAB-14240. This paper does not report original code. Source data is provided. Any additional information is available from the lead contact upon reasonable request.

Research involving human participants, their data, or biological material

Policy information about studies with human participants or human data. See also policy information about sex, gender (identity/presentation), and sexual orientation and race, ethnicity and racism.

Reporting on sex and gender	*Use the terms sex (biological attribute) and gender (shaped by social and cultural circumstances) carefully in order to avoid confusing both terms. Indicate if findings apply to only one sex or gender; describe whether sex and gender were considered in study design; whether sex and/or gender was determined based on self-reporting or assigned and methods used. Provide in the source data disaggregated sex and gender data, where this information has been collected, and if consent has been obtained for sharing of individual-level data; provide overall numbers in this Reporting Summary. Please state if this information has not been collected. Report sex- and gender-based analyses where performed, justify reasons for lack of sex- and gender-based analysis.*
Reporting on race, ethnicity, or other socially relevant groupings	*Please specify the socially constructed or socially relevant categorization variable(s) used in your manuscript and explain why they were used. Please note that such variables should not be used as proxies for other socially constructed/relevant variables (for example, race or ethnicity should not be used as a proxy for socioeconomic status). Provide clear definitions of the relevant terms used, how they were provided (by the participants/respondents, the researchers, or third parties), and the method(s) used to classify people into the different categories (e.g. self-report, census or administrative data, social media data, etc.) Please provide details about how you controlled for confounding variables in your analyses.*
Population characteristics	*Describe the covariate-relevant population characteristics of the human research participants (e.g. age, genotypic information, past and current diagnosis and treatment categories). If you filled out the behavioural & social sciences study design questions and have nothing to add here, write "See above."*
Recruitment	*Describe how participants were recruited. Outline any potential self-selection bias or other biases that may be present and how these are likely to impact results.*
Ethics oversight	*Identify the organization(s) that approved the study protocol.*

Note that full information on the approval of the study protocol must also be provided in the manuscript.

Field-specific reporting

Please select the one below that is the best fit for your research. If you are not sure, read the appropriate sections before making your selection.

☒ Life sciences ☐ Behavioural & social sciences ☐ Ecological, evolutionary & environmental sciences

For a reference copy of the document with all sections, see nature.com/documents/nr-reporting-summary-flat.pdf

Life sciences study design

All studies must disclose on these points even when the disclosure is negative.

Sample size	sample size was defined according to the maximum possible amount of cells expressing the transfected constructson microscopic slide
Data exclusions	No data was excluded in this study.
Replication	All experiments except H3.1 and H3.3 enrichment determination by ChIP-Seq have been replicated. The number of replicates is indicated in the text
Randomization	no randomization was needed for this study
Blinding	Blinding was performed during acquisition. Blinding could not be systematically performed during analysis since the output is fluorescent (TALE-HIRA-Clover constructs and TALE-Clover control) versus non-fluorescent non-transfected cells (NT) and/or localizing at chromocenters (TALE HIRA-Clover constructs) vs not localizing at chromocenters (TALE-Clover (Ctr)).

Reporting for specific materials, systems and methods

We require information from authors about some types of materials, experimental systems and methods used in many studies. Here, indicate whether each material, system or method listed is relevant to your study. If you are not sure if a list item applies to your research, read the appropriate section before selecting a response.

Materials & experimental systems

n/a	Involved in the study	
☐	☒	Antibodies
☐	☒	Eukaryotic cell lines
☒	☐	Palaeontology and archaeology
☒	☐	Animals and other organisms
☒	☐	Clinical data
☒	☐	Dual use research of concern
☒	☐	Plants

Methods

n/a	Involved in the study	
☐	☒	ChIP-seq
☐	☐	Flow cytometry
☐	☐	MRI-based neuroimaging

Antibodies

Antibodies used	H3.1/H3.2, Active motif #61629 (IgG2b)1/2000; H3.3 Active motif #91191 (IgG2a) 1/150; HA, Roche #1867423 1/2000; Aurora B, BD transduction laboratorie #611082 1/1000; HP1alpha, Sigma #H2164 1/1000 Oct 3/4 BD Bioscienes #611203; 1/1000 H4, Abcam #ab31830 1/1000; GFP in house made anti eGFP 1/100. This antibody list is also available in text
Validation	These antibodies have been already characterized in previous publications from our group or others.

Eukaryotic cell lines

Policy information about cell lines and Sex and Gender in Research

Cell line source(s)	KH2 mESCs (Beard, 2006 DOI: 10.1002/gene.20180), Suv39 dn mESCs {Lehnertz, 2003 DOI: 10.1016/S0960-9822(03)00432-9) and 3T3 (ATCC #CRL-1658) cells were used in this study
Authentication	None of the cells used were authenticated in this study
Mycoplasma contamination	cell lines used in this study were tested negative for mycoplasma contamination.
Commonly misidentified lines (See ICLAC register)	This study did not involve misidentified cell lines.

Plants

Seed stocks	*Report on the source of all seed stocks or other plant material used. If applicable, state the seed stock centre and catalogue number. If plant specimens were collected from the field, describe the collection location, date and sampling procedures.*
Novel plant genotypes	*Describe the methods by which all novel plant genotypes were produced. This includes those generated by transgenic approaches, gene editing, chemical/radiation-based mutagenesis and hybridization. For transgenic lines, describe the transformation method, the number of independent lines analyzed and the generation upon which experiments were performed. For gene-edited lines, describe the editor used, the endogenous sequence targeted for editing, the targeting guide RNA sequence (if applicable) and how the editor was applied.*
Authentication	*Describe any authentication procedures for each seed stock used or novel genotype generated. Describe any experiments used to assess the effect of a mutation and, where applicable, how potential secondary effects (e.g. second site T-DNA insertions, mosiacism, off-target gene editing) were examined.*

ChIP-seq

Data deposition

☒ Confirm that both raw and final processed data have been deposited in a public database such as GEO.

☐ Confirm that you have deposited or provided access to graph files (e.g. BED files) for the called peaks.

Data access links *May remain private before publication.*	Sequencing data from SNAP capture assays have been deposited in ArrayExpress under accession number E-MTAB-14240
Files in database submission	E-MTAB-14240.idf.txt<br>E-MTAB-14240.sdrf.txt

	H3.1 input mESC - ERS20585404.R1.fastq.gz, ERS20585404.R2.fastq.gz<br>H3.1 input NPC - ERS20585405.R1.fastq.gz, ERS20585405.R2.fastq.gz<br>H3.1 IP mESC - ERS20585406.R1.fastq.gz, ERS20585406.R2.fastq.gz<br>H3.1 IP NPC - ERS20585407.R1.fastq.gz, ERS20585407.R2.fastq.gz<br>H3.3 input mESC - ERS20585408.R1.fastq.gz, ERS20585408.R2.fastq.gz<br>H3.3 input NPC - ERS20585409.R1.fastq.gz, ERS20585409.R2.fastq.gz<br>H3.3 IP mESC - ERS20585410.R1.fastq.gz, ERS20585410.R2.fastq.gz<br>H3.3 IP NPC - ERS20585411.R1.fastq.gz, ERS20585411.R2.fastq.gz
Genome browser session (e.g. UCSC)	GRCm38, release 98

Methodology

Replicates	ChIP-seq for H3.1/H3.3 was performed in a single replicate in mESC or NPC and compared to publicly available data for QC
Sequencing depth	We performed PE100 sequencing on Illumina NovaSeq 6000 to at least 80M reads per sample.
Antibodies	SNAP capture beads (S9145S, NEB) were used to capture the H3.1/H3.3-SNAP
Peak calling parameters	We did not perform peak calling, but quantified reads at repeats. We used the GSAT_MM (n=72) from the ensembl repeat annotation.
Data quality	N/A
Software	Read mapping and preprocessing: bowtie2 v2.3.4.2 (Langmead, 2012), SAMtools v1.9 (Danecek, 2021)<br>Read quantification and plotting: python 3.9, pandas 1.5.3 (McKinney, 2010), numpy 1.23.5 (Harris et al., 2020), scipy 1.11.2 (Virtanen et al., 2020), matplotlib 3.6.2 (Hunter, 2007)

Flow Cytometry

Plots

Confirm that:

- [ ] The axis labels state the marker and fluorochrome used (e.g. CD4-FITC).
- [ ] The axis scales are clearly visible. Include numbers along axes only for bottom left plot of group (a 'group' is an analysis of identical markers).
- [ ] All plots are contour plots with outliers or pseudocolor plots.
- [ ] A numerical value for number of cells or percentage (with statistics) is provided.

Methodology

Sample preparation	*Describe the sample preparation, detailing the biological source of the cells and any tissue processing steps used.*
Instrument	*Identify the instrument used for data collection, specifying make and model number.*
Software	*Describe the software used to collect and analyze the flow cytometry data. For custom code that has been deposited into a community repository, provide accession details.*
Cell population abundance	*Describe the abundance of the relevant cell populations within post-sort fractions, providing details on the purity of the samples and how it was determined.*
Gating strategy	*Describe the gating strategy used for all relevant experiments, specifying the preliminary FSC/SSC gates of the starting cell population, indicating where boundaries between "positive" and "negative" staining cell populations are defined.*

- [ ] Tick this box to confirm that a figure exemplifying the gating strategy is provided in the Supplementary Information.

Magnetic resonance imaging

Experimental design

Design type	*Indicate task or resting state; event-related or block design.*
Design specifications	*Specify the number of blocks, trials or experimental units per session and/or subject, and specify the length of each trial or block (if trials are blocked) and interval between trials.*
Behavioral performance measures	*State number and/or type of variables recorded (e.g. correct button press, response time) and what statistics were used to establish that the subjects were performing the task as expected (e.g. mean, range, and/or standard deviation across subjects).*

Acquisition

Imaging type(s) | *Specify: functional, structural, diffusion, perfusion.*

Field strength | *Specify in Tesla*

Sequence & imaging parameters | *Specify the pulse sequence type (gradient echo, spin echo, etc.), imaging type (EPI, spiral, etc.), field of view, matrix size, slice thickness, orientation and TE/TR/flip angle.*

Area of acquisition | *State whether a whole brain scan was used OR define the area of acquisition, describing how the region was determined.*

Diffusion MRI ☐ Used ☐ Not used

Preprocessing

Preprocessing software | *Provide detail on software version and revision number and on specific parameters (model/functions, brain extraction, segmentation, smoothing kernel size, etc.).*

Normalization | *If data were normalized/standardized, describe the approach(es): specify linear or non-linear and define image types used for transformation OR indicate that data were not normalized and explain rationale for lack of normalization.*

Normalization template | *Describe the template used for normalization/transformation, specifying subject space or group standardized space (e.g. original Talairach, MNI305, ICBM152) OR indicate that the data were not normalized.*

Noise and artifact removal | *Describe your procedure(s) for artifact and structured noise removal, specifying motion parameters, tissue signals and physiological signals (heart rate, respiration).*

Volume censoring | *Define your software and/or method and criteria for volume censoring, and state the extent of such censoring.*

Statistical modeling & inference

Model type and settings | *Specify type (mass univariate, multivariate, RSA, predictive, etc.) and describe essential details of the model at the first and second levels (e.g. fixed, random or mixed effects; drift or auto-correlation).*

Effect(s) tested | *Define precise effect in terms of the task or stimulus conditions instead of psychological concepts and indicate whether ANOVA or factorial designs were used.*

Specify type of analysis: ☐ Whole brain ☐ ROI-based ☐ Both

Statistic type for inference | *Specify voxel-wise or cluster-wise and report all relevant parameters for cluster-wise methods.*

(See Eklund et al. 2016)

Correction | *Describe the type of correction and how it is obtained for multiple comparisons (e.g. FWE, FDR, permutation or Monte Carlo).*

Models & analysis

n/a	Involved in the study
☐	☐ Functional and/or effective connectivity
☐	☐ Graph analysis
☐	☐ Multivariate modeling or predictive analysis

Functional and/or effective connectivity | *Report the measures of dependence used and the model details (e.g. Pearson correlation, partial correlation, mutual information).*

Graph analysis | *Report the dependent variable and connectivity measure, specifying weighted graph or binarized graph, subject- or group-level, and the global and/or node summaries used (e.g. clustering coefficient, efficiency, etc.).*

Multivariate modeling and predictive analysis | *Specify independent variables, features extraction and dimension reduction, model, training and evaluation metrics.*
